# Transcription factor abundance controlled by an auto-regulatory mechanism involving a transcription start site switch

**DOI:** 10.1093/nar/gkt1136

**Published:** 2013-11-14

**Authors:** Richard Patryk Ngondo, Philippe Carbon

**Affiliations:** Architecture et Réactivité de l’ARN, Université de Strasbourg, CNRS, IBMC, 15 Rue René Descartes, 67084 Strasbourg, France

## Abstract

A transcriptional feedback loop is the simplest and most direct means for a transcription factor to provide an increased stability of gene expression. In this work performed in human cells, we reveal a new negative auto-regulatory mechanism involving an alternative transcription start site (TSS) usage. Using the activating transcription factor ZNF143 as a model, we show that the ZNF143 low-affinity binding sites, located downstream of its canonical TSS, play the role of protein sensors to induce the up- or down-regulation of ZNF143 gene expression. We uncovered that the TSS switch that mediates this regulation implies the differential expression of two transcripts with an opposite protein production ability due to their different 5′ untranslated regions. Moreover, our analysis of the ENCODE data suggests that this mechanism could be used by other transcription factors to rapidly respond to their own aberrant expression level.

## INTRODUCTION

Maintaining the adequate gene expression pattern in cells is an essential feature of multicellular complexity and diversity. This regulation is achieved through multiple factors that act at the transcriptional and/or post-transcriptional level. The major regions involved in this regulation are untranslated regions of messenger RNAs (5′ UTRs and 3′ UTRS), introns and *cis*-regulatory regions on DNA (1). Alternative promoter usage and alternative splicing largely contribute to the generation of transcripts that are differentially regulated (1–3). These transcripts could have diverse exons composition and/or contain altered 5′ UTRs and 3′ UTRs (1,4,5).

Auto-regulation is one of the simplest and the most efficient regulatory mechanism used by the cell to maintain the proper gene expression at multiple levels. Good examples are RNA-binding proteins such as TDP-43 (6,7) and HuR (8) that regulate their expression by targeting and affecting the stability of their own mRNA. At the transcriptional level, auto-regulatory loops are highly conserved in vertebrate evolution (9) and are well described from the lambda phage to more complex higher eukaryotes (10). The principal benefit of such mechanisms is to restore rapidly and efficiently the homeostasis of proteins in the cells. In this regard, it has been demonstrated that the negative auto-regulation of transcription factors speeds up the response times of transcription networks and provides stability by limiting the range over which the concentrations of network components fluctuate (11,12). Transcriptional auto-regulation can be mediated by direct interactions as for BRCA1 (13) or indirectly as for the auto-regulatory network of p53 (14). Another well-described complex transcriptional auto-regulatory network involves OCT4, SOX2 and NANOG pluripotency transcription factors in embryonic stem cells (15–17). To date, only few direct transcriptional feedback loops have been reported including PAX4, Hes1, BRCA1, HNF4α, OCT4, SOX2 and NANOG (13,15,18–20). However, the mechanistic insights of such regulations have not been fully explored. Taking in consideration the vast and functionally varied transcription factor population in human (21), as well as their potential involvement in feedback loops (9), we could expect a wide diversity of transcriptional auto-regulatory mechanisms.

ZNF143 is a zinc-finger transcription factor regulating dozens of non-coding genes and more than 3000 protein coding genes (22). Targeted genes are essentially involved in rapid cell proliferation (22,23), are required for the normal development (24) and are important to the self-renewal and maintenance of embryonic stem cells (25–27). It has been previously shown that ZNF143, also known as Staf, is required for the normal expression of the essential tRNA^Sec^ gene (28–30). ZNF143 has a vertebrate-specific paralog called ZNF76 (31). The two proteins that share 63% of identity, have exactly the same DNA-binding domain (DBD), are both able to activate transcription and have the same binding profile *in vivo* (22,31). Both repressor and activator functions have been attributed to ZNF76 (31,32), however, its role *in vivo* remains unknown.

To date, nothing was known about the transcriptional mechanisms underlying the expression of ZNF143. In this study, we present evidence of a feedback loop mechanism that acts at the transcriptional level to regulate ZNF143 expression. We demonstrate that a transcriptional activator can negatively regulate its own expression when over-expressed in the cell. In addition, this study represents the first evidence of a transcriptional auto-regulatory mechanism, relaying on the use of non-canonical binding sites, as sensors to trigger a transcriptional start site (TSS) switch. Finally, we hypothesize that this mechanism could certainly represent a more general transcriptional auto-regulation strategy.

## MATERIALS AND METHODS

### Antibodies and constructs

Antibodies and constructs used in this study are described in Supplementary Materials and Methods.

### Cell lines and induced protein expression and knockdown

All the cell lines were maintained in standard DMEM medium supplemented with 10% Fetal Calf Serum and 1% Pen-Step (GIBCO). The FLP143, FLP143-HA, FLP_THAP11-HA and FLP76 inducible stable cell lines were obtained and maintained as described in (22). The FLP143-EGR2 cell line was obtained as described in (22) using the pGMC plasmid (see Supplementary Materials and Methods) expressing a chimeric ZNF143 protein (ZNF143-EGR2) wherein the seven zinc fingers DBD was substituted by that of EGR2 (also known as KROX20) (31) composed of three zinc fingers. Protein expression was induced by the addition of 1 µg/ml of doxycycline in the culture medium. The SH1_7 stable cell line was obtained after the puromycin selection (5 µg/ml) of T-rex 293 cells transduced with PTRIPZ lentiviral inducible shRNAmir V3THS_308811 (Thermo Scientific) targeting ZNF143 transcript. The knockdown was induced by the addition of 1 µg/ml of doxycycline in the culture medium.

### RNA preparation and expression analysis

The ectopic gene expression in the stable cell lines was induced with doxycycline, and the cells were collected at different time points post-induction. Total RNA was extracted using TRI-REAGENT (Euromedex), and Poly(A)+ RNA was enriched from total RNA using µMACS mRNA Isolation Kit (Miltenyi Biotec). The cDNA obtained by reverse transcription with random primers (dN9) was amplified with specific primers on the CFX96 Touch™ Real-Time PCR Detection System (Bio-Rad) using EvaGreen qPCR Mix Plus (Euromedex). Primer sequences are available in Supplementary Table S1. All reactions were carried out in triplicate. The relative expression ratio was calculated using the CFX Manager™ Software (Bio-Rad). The tissue qPCR arrays gene expression analysis was performed on human disease tissues (TissueScan™ Cancer Survey Tissue qPCR Panel 96 – I CSRT101, OriGene) and on mouse tissues (TissueScan™ Mouse developmental Tissue qPCR Panel I MDRT101, OriGene). The cDNAs from human and mouse qPCR arrays were normalized against ß-actin and GAPDH levels, respectively. The relative expression was calculated using the ΔΔ*C*_t_ method.

### Protein preparation and western-blot analysis

Cells were collected at different time points post-induction and lysed. The proteins from the lysate were separated on an 8% SDS–PAGE and subjected to western-blot analysis using antibodies against ZNF143, α-tubulin and TBP. The western-blot signal quantification was performed on Quantity One Software (Bio-Rad).

### Chromatin immunoprecipitation assay

Chromatin immunoprecipitation (ChIP) assays were performed as previously described (22). The average fragment size of the sheared DNA is of 200–400 bp. After DNA purification, enrichment analysis was performed by quantitative real time PCR (qPCR). qPCR was performed in triplicates on a CFX96 Touch™ Real-Time PCR Detection System (Bio-Rad) using EvaGreen qPCR Mix Plus (Euromedex) and input DNA as the standard. The input DNA was diluted 500 times compared with the ChIPed DNA to measure the enrichment of specific genomic regions relative to the negative control regions devoid of the studied binding sites. Enrichment was determined by the ΔΔ*C*_t_ method. Primer sequences are available in Supplementary Table S1.

### Northern blot

FLP143 or SH1_7 cells were treated with 1 µg/ml of doxycycline 48 h prior the transfection performed using Lipofectamine™ 2000 (Invitrogen) according to the manufacturer’s instructions. The total RNA was isolated 24 h post-transfection and treated with recombinant DNase I (Roche). RNA samples [20–30 µg of total RNA or 5 µg of poly(A)+ enriched RNA] were loaded on 1% formaldehyde agarose gels, transferred onto a Hybond N^+^ nylon membrane (Amersham Biosciences), UV crosslinked (UV Stratalinker 1800, Stratagene) and probed with internally ^32^P labeled RNA or 5′ ^32^P labeled oligodeoxynucleotides. RNA probes were synthesized by *in vitro* T7 transcription with α^32^P labeled ATP using specific PCR templates. The sequences of the primers used to amplify the PCR templates are available in Supplementary materials (Supplementary Table S1). The prehybridization and hybridization were carried out using PerfectHyb™Plus Solution (Sigma) at 42°C and the washes performed according to the supplier’s recommendations. Transcripts were visualized and quantified with a Fujifilm Bio-Imaging Analyzer System.

### Luciferase assay

FLP143 and SH1_7 cells were induced or not induced with doxycycline 48 h prior the transfection in 96 wells plates with the different luciferase constructs. The luciferase assay was performed 24 h post-transfection using the Dual*-*Luciferase® Reporter Assay System (Promega) according to the manufacturer recommendations. The luminescence signal was measured on a GloMax® 96 Microplate Luminometer (Promega). Each essay was performed in three biological replicates, and the *Renilla* luciferase signal was normalized to the firefly luciferase activity.

### 5′ RACE-PCR

The 5′ RACE-PCR experiment was performed using the FirstChoice® RLM-RACE Kit (Life Technologies) according to the manufacturer recommendations on total RNA extracted from FLP143 cells treated or not with 1 µg/ml of doxycycline. The endogenous ZNF143 cDNA was targeted with two gene-specific inner/outer PCR primer pairs: PN627/PN628 and PN625/PN626 (Supplementary Table S1). The cDNA from transfected pNG107 construct was targeted with two specific inner/outer PCR primer pairs: PN270/PN500 and PN270/PN640 (Supplementary Table S1). The nested-PCR products were analyzed on 2% agarose gel and sequenced to identify the 5′ end of the targeted mRNA.

### Electromobility shift assay

Recombinant ZNF143 DBD was produced using the GST gene fusion system as previously described (22). The DNA fragments containing the SBS/A, SBS/B, mut SBS/B−, SBS/C and mut SBS/C− binding sites were obtained by annealing two complementary oligonucleotides 5′ ^32^P-labeled (Supplementary Table S1). Binding assays were performed essentially as described (33) with 20 fmol of labeled probe. Protein–DNA complexes were resolved by electrophoresis on 4% native polyacrylamide gels containing 0.25× Tris-borate-EDTA. Competition was performed with a 200-fold molar excess of unlabeled specific competitor or unspecific competitor.

### Determinations of relative binding affinities

Quantitative competitive electromobility shift assay (EMSA) was used to compare the relative affinities of the ZNF143-binding sites as described previously (34). Briefly, the recombinant ZNF143 DBD was incubated for 20 min at room temperature with zero and increasing concentrations of the unlabeled oligonucleotide duplex probes containing SBS/B, SBS/C and optimal SBS site from the BUB1B promoter (33). The labeled probe containing the optimal SBS site was then added to a final volume of 10 µl. Following electrophoresis, the bound probe was quantified (Using Fujifilm Bio-Imaging Analyzer System), and the fraction of maximal binding at each competitor concentration was calculated as the ratio of bound probe plus competitor to bound probe with no competitor. An unspecific competitor, devoid of ZNF143-binding site, has been used as a negative control. A curve was then fitted to the values for the fraction of maximal binding at known competitor DNA concentrations.

### ChIP-seq data

In this study, we used the ZNF143 ChIP-seq data from our previous study (Geo access: GSE39263) (22) and Encode ChIP-seq data on TAF1 and TBP in HeLa cells as well as on 87 other sequence-specific transcription factors (35).

## RESULTS

### Induced ectopic ZNF143 over-expression downregulates the endogenous ZNF143 expression

**Figure 1.**
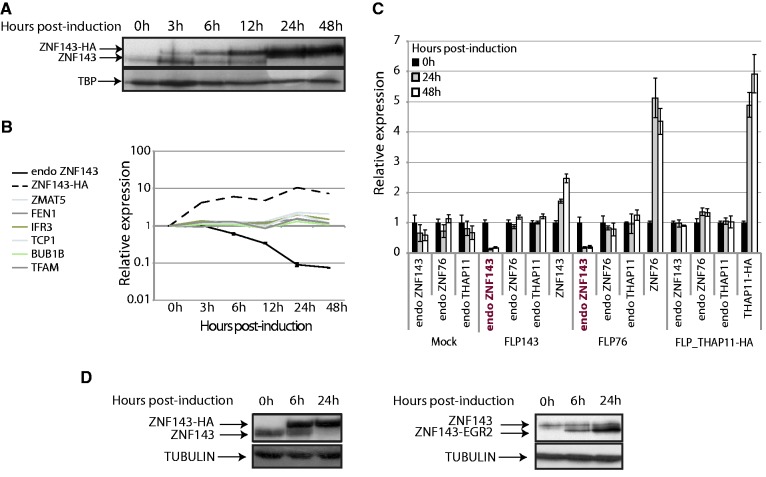
The over-expression of a ZNF143 and ZNF76 transgene specifically represses the expression of the endogenous ZNF143 gene. **(A)** Western blotting performed with ZNF143 antibody on total protein extracts from FLP143HA cells at different times points (in h) after induction of the expression of the ZNF143-HA protein. The arrows indicate the endogenous ZNF143 (68.8 kDa) and the tagged ZNF143-HA (72.4 kDa) proteins. The TBP protein is used as loading control. **(B)** Gene expression measured by RT-qPCR on total RNA extracted from FLP143HA cells at different time points (in h) post-induction with doxycycline. The relative expression of the ZNF143 endogenous mRNA (endo ZNF143) was followed using primers specific to the 5′ UTR region. The transgene ZNF143-HA, ZMAT5, FEN1, IFR3, TCP1, BUB1B and TFAM were also followed over the same time course. The relative expression was normalized to both the TBP and UBC gene expression levels. The error bar for the endo ZNF143 expression is the standard deviation of two biological replicates. **(C)** Gene expression measured by RT-qPCR on total RNA extracted from FLP-mock (control) and from FLP143, FLP76, FLP_THAP11-HA stable cell lines at 0 (black), 24 (gray) and 48 h (white) post-treatment with doxycycline. The relative expression of each endogenous gene (endo ZNF143, endo ZNF76 and endo THAP11) and transgene (ZNF76, ZNF143 and THAP11-HA) for each cell type is represented on the *x*-axis. The expression is normalized to the GAPDH levels, and the error bar is the standard deviation of three replicates. **(D)** The endogenous ZNF143 protein expression was followed by western blot, after the over-expression of a chimeric ZNF143 with impaired DNA-binding specificity. The chimeric ZNF143-EGR2 protein was obtained by substituting the DBD of ZNF143 by that of EGR2 (KROX20) protein. Stable cell lines expressing the tagged (ZNF143-HA) or the chimeric (ZNF143-ERG2) proteins were induced for 6 and 24 h with doxycycline. The resulting protein extract was analyzed by western blot with anti-ZNF143 antibodies. The arrows indicate the endogenous ZNF143 (68.8 kDa), the tagged ZNF143-HA (72.4 kDa) and the chimeric (57.72 kDa) proteins. The α-tubulin is used as loading control.

We generated stable cell lines expressing an HA-tagged (FLP143-HA) or a non-tagged ZNF143 protein (FLP143) under the control of an inducible promoter. Interestingly, we observed that the doxycycline-induced ectopic expression of ZNF143-HA resulted in a decrease in the endogenous ZNF143 protein level as seen by western blot (Figure 1A, take in account the TBP signal). The production of the ZNF143-HA protein is detectable already 3 h post-induction, and its level rapidly increases over the time. In contrast, the endogenous ZNF143 protein that is distinctly detected up to 12 h disappears 24 h post-induction of the transgene expression (Figure 1A). The down-regulation of the endogenous ZNF143 is observed not only at the protein level but also at the mRNA level. We followed specifically by RT-qPCR the endogenous ZNF143 mRNA using primers that target the 5′ UTR, absent in the induced gene. The transgenic mRNA was followed using primers targeting specifically the HA-tag. After induction, we observed that the ZNF143-HA mRNA level rapidly increases by 10 times after 24 h (Figure 1B). At the same time, the endogenous ZNF143 mRNA level is decreased 6 h post-induction and is declining continuously by more than 10 times after 24–48 h (Figure 1B). In comparison, the expression of known ZNF143 target genes ZMAT5, FEN1, IRF3, TCP1, BUB1B and TFAM (33,36–39) is increased up to two times after 24 h (Figure 1B). This drastic ZNF143 down-regulation was also confirmed by northern-blot analysis of ZNF143 mRNA in FLP143 cells over-expressing a non-tagged version of ZNF143 (Supplementary Figure S1A). Moreover, we observed that this phenomenon is reversible. Indeed, after the inductor withdrawal in FLP143 cells, the endogenous ZNF143 expression level returned to normal both at the protein and mRNA levels (Supplementary Figure S1B and C).

### ZNF143 down-regulation is also induced by ZNF76 over-expression

To test if the aforementioned phenomenon is restricted to ZNF143, we monitored the endogenous expression of two other transcription factors following the over-expression of their analogous transgenes. For this purpose, we used FLP76, FLP_THAP11-HA, FLP143 and FLP-mock (control) inducible stable cell lines over-expressing, respectively, ZNF76, THAP11-HA, ZNF143 and no-protein. ZNF76 was previously characterized as the ZNF143 paralog with the same DNA-binding specificities (31). THAP11 in this assay was used as a control. Gene expression analysis was performed before induction with doxycycline and 24–48 h post-induction (Figure 1C). Total ZNF143, ZNF76 and THAP11 mRNA levels were highly increased after induction in FLP143, FLP76 and FLP_THAP11-HA cell lines, respectively. The doxycycline induction had no effect on the endogenous ZNF143, ZNF76 and THAP11 expression in the empty control stable cell line (Mock). THAP11-HA over-expression had as well no impact on ZNF143, ZNF76 and THAP11 levels. Surprisingly, alike ZNF143, ZNF76 over-expression caused a 10-time fold down-regulation of the endogenous ZNF143. However, the level of the endogenous ZNF76 mRNA was not affected by any of the proteins. This ZNF76 mediated down-regulation of ZNF143 was also observed at the protein level (data not shown), suggesting that the DNA-binding specificities of ZNF143 and ZNF76 mediate the observed regulation. To endorse this assumption, we generated a stable cell line expressing a chimeric ZNF143 with an altered DBD. The chimeric protein was obtained by substituting the DBD of ZNF143 by that of the EGR2 protein, also known as KROX20 (31). After the induction of the expression of the ZNF143-EGR2 protein, we see no changes in the expression of the endogenous ZNF143 (Figure 1D, right panel). The level of the chimeric protein is increased 6 and 24 h after induction, while the level of the endogenous ZNF143 stays constant. In contrast, the induction of the HA-tagged ZNF143 protein causes a specific down-regulation of its endogenous counterpart (Figure 1D, left panel). We conclude that the DBD of ZNF143 is therefore required for the observed down-regulation of the endogenous protein.

### ZNF143 binds its own minimal promoter region

**Figure 2.**
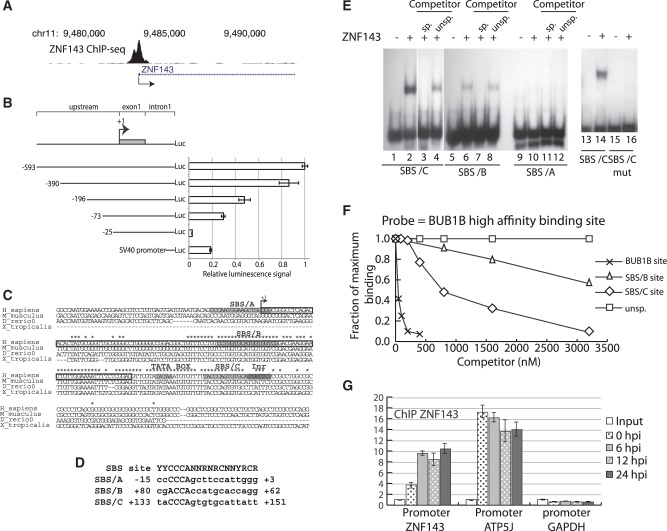
ZNF143 occupies its own minimal promoter region. **(A)** UCSC genome browser view of a ZNF143 ChIP-seq peak (22) located on its own promoter region. **(B)** Identification of the 5′ minimal region required for the activity of ZNF143 promoter. Left part, schematic representation of the constructs containing the shortened upstream promoter regions, the first non-coding exon (112 bp) and the truncated ZNF143 first intron (302 bp), in front of the *Renilla* luciferase gene (Luc). The truncated intron contains the 5′-part (+113/+234) linked to the 3′-part (+10170/+10344) of the intron. The shortened upstream regions are indicated in base pairs relative to the ZNF143 TSS (+1). The numbering (+1) starts from the first nucleotide of the RefSeq sequence NM_003442. The psiCHECK-2 vector containing the SV40 promoter is used as a reference. Right part, relative *Renilla* luciferase activity of each construct normalized to the firefly luciferase activity. The error bar corresponds to the standard deviation of three biological replicates. **(C)** Multiple sequence alignment of the ZNF143 region spanning the TSS in human (H_sapiens), mouse (M_musculus), zebra fish (D_rerio) and xenope (X_tropicalis). The conserved nucleotides are indicated with a star. An arrow (+1) indicates the TSS; the first non-coding exon is framed in black. The potential ZNF143-binding sites (SBS/A, SBS/B and SBS/C), a TATA box-like and an initiator (Inr) element are highlighted in gray. **(D)** Comparison of the SBS/A, SBS/B, SBS/C and SBS consensus sequence (42), (Y, N and R stand for T/C, any nucleotide and A/G, respectively). **(E)** Electrophoretic mobility shift assay performed with the ^32^P labeled oligonucleotides probes corresponding to the putative ZNF143-binding sites: SBS/A (lanes 9–12), SBS/B (lanes 5–8), SBS/C (lanes 1–4) and the mutated SBS/C (CCCA substituted to AAAC, lanes 13–16). Probes were incubated in the absence (lanes 1, 5, 9, 13 and 15) or presence of ZNF143 DBD (lanes 2, 3, 4, 6, 7, 8, 10, 11, 12, 14 and 16). Reactions were performed in the presence of a 1000-fold molar excess of unlabeled specific competitor (sp.) (lanes 3, 7 and 11) and unspecific competitor (unsp.) (lanes 4, 8 and 12). **(F)** Measurements of the relative binding affinities of the ZNF143-binding domain to the SBS/C, SBS/B and the optimal SBS site from BUB1B promoter (33). Relative affinities of different sequences were determined by comparing their effectiveness as binding competitors. Various concentrations of unlabeled oligonucleotides containing the sites were pre-incubated with equal concentrations of ZNF143 DBD. A constant amount of labeled probe containing the optimal sequence (from BUB1B promoter) was subsequently added. After the binding equilibrium was reached, the extent of competition was analyzed following gel electrophoresis as described in ‘Materials and Methods’ section. An unspecific (unsp) competitor, devoid of ZNF143-binding site, has been used as a negative control. **(G)** ChIP experiment with ZNF143 antibodies performed on FLP143 cells: before induction of ZNF143 over-expression with doxycycline (0 hpi), 6, 12 and 24 h post-induction (hpi). The enrichments were measured by qPCR using primers specific to regions located: 150 bp upstream the ZNF143 TSS (+1)(ZNF143 promoter), in the ATP5J promoter region containing an SBS site and in the GAPDH promoter region devoid of SBS site. The enrichment was calculated compared to a non-specific region located 1 kb downstream of the ZNF143 TSS and using the input DNA as a standard. The error bar corresponds to the standard deviation of three replicates.

The accumulation of ZNF143 after the induction of FLP cell lines suggested that the ZNF143 transgene, unlike the endogenous ZNF143, was not subjected to a negative feedback loop. From the ChIP-seq data generated in our previous study (22), we identified a ZNF143-binding event, centered on the middle part of the ZNF143 first exon, in all human and mouse cell lines tested (Figure 2A; Supplementary Figure S2A). The presence of this peak suggests the possibility of an auto-regulatory feedback in the control of ZNF143 transcription. As a prerequisite to the investigation of this phenomenon, we defined in a first attempt the ZNF143 minimal promoter. We engineered a *Renilla* luciferase reporter construct headed by the 593 nt located upstream of the TSS, the first exon (+1/+112) and the shortened ZNF143 first intron (5′-part: +113/+234 associated with the 3′-part: +10170/+ 10344) (Figure 2B). We transiently transfected 293 T-rex cells with the luciferase reporter constructs containing a progressively shortened 5′ upstream regions and measured the luciferase activity of the resulting cell extracts (Figure 2B). The highest luciferase activity was observed for the largest construct −593. The activity decreased progressively to 30% for the −73 construct and was almost abolished for the shortest −25 construct. The region, essential to maintain significant transcriptional activity, was located between the −73 and −25. This allowed us to restrict our study to a region covering positions −73 bp to +234 relative to the TSS. Surprisingly, this minimal region contains a 98-bp element (+54/+151) with a high degree of sequence conservation in vertebrates (Figure 2C), located in the first non-coding exon and in the beginning of the adjacent intron. Such conservation of non-coding gene elements suggests a putative important regulatory role for this region. Computational analysis of the −75/+234 region with the MatInspector software (40) revealed the presence of interesting motifs. The conserved intronic region contains a TATA-box like element (Figure 2C, position + 120 relative to TSS) and an Initiator element (Inr, TTATTC position +147) located 25 bp after the TATA-box (Figure 2C). ChIP-Seq data from the ENCODE consortium suggests strongly that the TATA-box and the Inr element are bound by TBP and TAF1, respectively (Supplementary Figure S2B). The region −75/+234 also contains three potential ZNF143-binding motifs: SBS/A (position −15), SBS/B (position +80) and SBS/C (position +133), with the SBS/B and SBS/C being conserved in vertebrates genomes (Figure 2C). The three SBS matched the consensus ZNF143-binding motif with a relatively low score (A: 0,800; B: 0,797; C: 0,823, respectively) (Figure 2D), likely reflecting a non-optimal binding of ZNF143 at these sites (34). By performing EMSA, we found that the ZNF143 DBD (Figure 2E, lanes 2–4 and lanes 6–8) binds specifically the SBS/B (located in the non-coding exon) and SBS/C (located in the intron) but not the SBS/A (Figure 2E, lane 10). In addition, point mutations in critical positions 3–6 (34) of the SBS/B and C motifs, completely abolish the ZNF143 binding *in vitro*, as illustrated in Figure 2E for SBS/C (compare lanes 14 and 16). The relative affinities of the SBS/B and SBS/C sites compare to an optimal binding site [taken from BUB1B promoter (33)] were evaluated by *in vitro* quantitative competitive binding assays (Figure 2F). The relative capacities of increasing concentrations of the SBS/B, SBS/C and the optimal site to compete for the binding to the ZNF143 with a constant concentration of the labeled optimal site were assessed. The ZNF143-DNA binding competition assay convincingly demonstrates that in our experimental conditions, both the SBS/B and SBS/C sites have a lower binding capacity to ZNF143 compared to an optimal binding site (BUB1B site). Indeed, at a concentration inhibiting 50% of the maximal binding, ZNF143 binds about 18 and 80 times more tightly the optimal sequence than to the SBS/C and SBS/B motifs, respectively. We next examined the ZNF143 occupancy on its own promoter region by ChIP-qPCR after ZNF143 over-expression (Figure 2G). The FLP143 cells over-expressing ZNF143 were used for ChIP experiments with antibodies specific to ZNF143 at different time points post-induction. The ChIP enrichment relative to the control regions is increased by more than two times on the ZNF143 promoter after its own ectopic over-expression (Figure 2G). On the other hand, ZNF143 occupancy did not vary on the ATP5J promoter (Figure 2G) containing an SBS consensus motif (22). This ZNF143 amplified binding to its promoter region is most likely a reflection of the lower affinity of ZNF143 to SBS/B and SBS/C sites.

### A ZNF143 transcript variant is initiated in the first canonical intron

**Figure 3.**
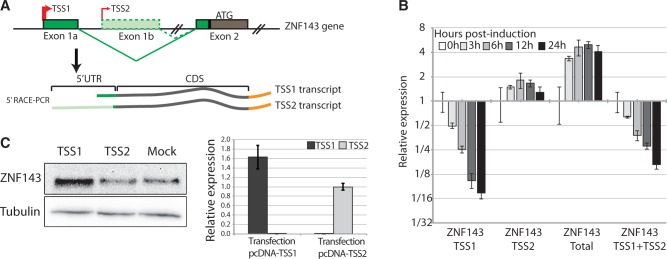
ZNF143 negative auto-regulation involves the down-regulation of its most abundant TSS1 transcript. **(A)** Schematic representation of the ZNF143 gene and of the two transcripts (TSS1 and TSS2 transcripts) identified by 5′ RACE PCR in total RNA from both induced and non-induced FLP143 cells. The TSSs are represented with arrows. The TSS1 transcript initiated at TSS1 contains the first non-coding exon (Exon1a) spliced to the second exon (Exon2) containing the initiation codon ATG. The TSS2 transcript initiated at TSS2 in the canonical first intron includes an alternative first non-coding exon (Exon 1 b) and other alternative non-coding exons (not shown) also spliced to the canonical second exon (Exon2). **(B)** Relative ZNF143 gene expression measured by qRT-PCR from total RNA extracted from FLP143 cells at multiple time points (in h) post-induction with doxycycline. The expression of the two endogenous ZNF143 alternative transcripts (ZNF143 TSS1 or ZNF143 TSS2), the total (endogenous and transgene) ZNF143 transcripts (ZNF143 Total) and the total endogenous transcripts (ZNF143 TSS1+TSS2) were measured using specific primers located, respectively, in the Exon1a, Exon1b, the CDS and 3′ untranslated region (Supplementary Table S1). The expression is normalized to the GAPDH levels, and the error bar is the standard deviation of three replicates. **(C)** 293 T-rex cells were transfected with pcDNA 3.1 constructs expressing the full-length TSS1 or TSS2 cDNAs. As a control, the cells were transfected with the empty pcDNA3.1 vector (mock). Left panel, western-blot analysis of ZNF143 protein level 48 h post-transfection in the control cells (mock) or after over-expression of the TSS1 or the TSS2 transcripts. Right panel, relative expression of TSS1 and TSS2 transcripts, measured by RT-qPCR, in total RNA from cells transfected with the TSS1 and TSS2 constructs.

Taken together, all the observations described above suggest a putative regulation of ZNF143 expression at the transcriptional level. In this regard, we first examined by ChIP-qPCR the presence of RNA polymerase II (Pol II) and histone modification marks (H3K4me3 and H3K9me3) on the ZNF143 promoter region before and 24 h post-induction (Supplementary Figure S3). We did not observe any enrichment for H3K9me3 repression marks. On the other hand, we obtained similar Pol II enrichments before and after induction, showing that this region stays in a transcriptionally active state. Histone H3K4me3 activation mark showed a slightly lower enrichment 24 h post-induction, suggesting a different chromatin context. In a second step, we mapped the TSS of ZNF143 by 5′ RACE-PCR on total RNA extracted from FLP143 cells before and after induction. Two families of TSS were identified in non- and 24 h-induced conditions (Figure 3A; Supplementary Figure S4A). The first family (TSS1) is located in a 90-bp window close to the reference TSS (NM_003442). The second family (TSS2) is located at 148–149 bp from the reference TSS, in the putative Inr element at the beginning of the first intron (Figure 2C; Supplementary Figure S4A). These 5′ RACE-PCR data are supported by the presence of two populations of expressed sequence tags (41) one initiated in the TSS1 window and the other (TSS2) in the ZNF143 first intron (Supplementary Figure S4B). The sequencing of a cDNA clone H04D117B14 (obtained from the RIKEN human cDNA library) initiated at the TSS2, combined with the 5′ RACE-PCR results, clearly evidenced the presence of an alternative first non-coding exon (Exon 1 b) (Figure 3A; Supplementary Figure S4C). The two populations of ZNF143 transcripts (TSS1 and TSS2 transcripts) (Figure 3A; Supplementary Figure S4) have exactly the same coding sequence (CDS) but have strikingly different 5′ UTRs. The canonical TSS1 transcript has a 119 nt long 5′ UTR composed of one non-coding exon, whereas the alternative TSS2 transcript has a 1222 nt long 5′ UTR composed of four non-coding exons (Supplementary Figure S4C). Only a 7-bp region located in 3′ of the 5′ UTRs, coming from the coding exon, is common to both transcripts (Supplementary Figure S4C). Taking advantage of the differences in 5′ UTR, we specifically followed both TSS1 and TSS2 transcripts by RT-qPCR in cells over-expressing or not ZNF143. Before induction, we noted the considerable difference in the number of cycles required for the TSS1 and TSS2 transcripts detection (Supplementary Figure S4D). Considering the six cycles difference, the TSS2 transcript is around 20 times less present than the TSS1 transcript before ZNF143 over-expression. After the induction of FLP143 cells, the total level of ZNF143 mRNA in the cell goes up by four times already after 6 h (Figure 3B), while the endogenous ZNF143 mRNA (TSS1 + TSS2) goes progressively down by eight times after 24 h (Figure 3B). However, if we look distinctly at the TSS1 and TSS2 transcripts we observe that the TSS1 transcript level drops down drastically by more than 10 times after 24 h, while the TSS2 transcript level stays constant or is slightly increased (Figure 3B). To test if the TSS2 transcript was prone to produce the ZNF143 protein, we transfected 293 T-rex cells, with plasmids expressing the TSS1 or TSS2 full-length cDNA, and followed the protein expression by western blot (Figure 3C). The over-expression of the TSS1 transcript in the cells, as expected, resulted in higher ZNF143 protein level compared to the mock-transfected cells (Figure 3C). In contrast, the over-expression of the TSS2 transcript does not result in significant production of the ZNF143 protein compared to the control cells (Figure 3C).

Taken together these observations show that ZNF143 over-expression induces a TSS switch, from one generating the canonical TSS1 transcript to another one producing a less abundant and untranslated TSS2 transcript.

### ZNF143 auto-regulation involves a TSS switch dependent on non-canonical SBS sites

**Figure 4.**
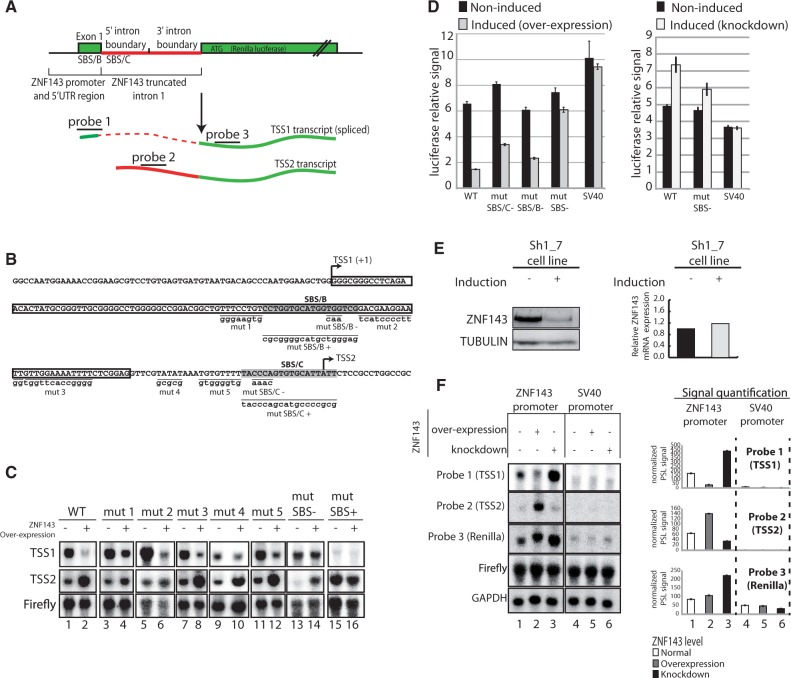
ZNF143 transcriptional auto-regulation involves a TSS switch mechanism using non-canonical SBS sites. **(A)** Schematic representation of the used heterologous system. The construct contains the region upstream (73 bp) of the ZNF143 TSS, the first non-coding exon (Exon1) and the splicing competent truncated ZNF143 first intron fused to the CDS of the *Renilla* luciferase. The spliced TSS1 is indicated in green and dashed red. The TSS2 transcript is not spliced and is indicated in plain green and red. The probes targeting the first exon (probe 1), the intron (probe 2) and the CDS of the *Renilla* luciferase (probe 3) are depicted. **(B)** Sequence of the ZNF143 promoter region cloned in the reporter construct. The TSSs, canonical (TSS1) and alternative (TSS2) are represented with an arrow. The ZNF143 first non-coding exon is framed, and both SBS/B and SBS/C sites are highlighted in gray. All the mutated positions are underlined, and the substituted sequences are indicated in lowercase. SBS/B− and SBS/C− correspond to mutations in the SBS site. SBS/B+ and SBS/C+ are creating high affinity consensus ZNF143-binding sites (42). The mutations mut 1 to mut 5 correspond to mutation in other regions of the conserved sequence block (Figure 2C). **(C)** Northern-blot analysis of the transcripts derived from transfected indicated constructs in FLP143 cells before (−) and after (+) 48 h doxycycline induction. The constructs are carrying the mutations indicated in (B). The mut SBS− and the mut SBS+ construct bear both the mut SBS/B and mut SBS/C mutations indicated in (B). All the constructs express the firefly luciferase gene under the control of a HSV-TK promoter. TSS1, TSS2 transcripts, Firefly luciferase CDS and GAPDH mRNA were detected with specific probes depicted in (A) for TSS1 and TSS2 transcripts. Firefly signal is used as an internal standard to normalize to *Renilla* signal and GAPDH signal serves as a loading control. **(D)** Luciferase assay performed with the different reporter constructs in ZNF143 over-expression and knockdown conditions. Left panel, relative *Renilla* luciferase activity in non-induced and induced (over-expression) FLP143 cells transfected with the WT construct, mutated in SBS/C (mut SBS/C−), mutated in SBS/B (mut SBS/B−) and mutated in both SBS/C and SBS/B (mut SBS−). SV40 promoter is used as negative control. The *Renilla* luciferase signal was normalized to the firefly luciferase signal. Right panel, *Renilla* luciferase activity signal in non-induced and induced (knockdown) SH1_7 cells, with WT and mut SBS−. **(E)** ZNF143 expression measured by western blot (left panel) and RT-qPCR (right panel) in SH1_7 stable cell line before (−) and 72 h post-induction (+) with doxycycline. Tubulin levels serves as a loading control for the western blot. ZNF143 mRNA levels are normalized to TBP and UBC expression levels. **(F)** Northern-blot analysis of the transcripts derived from the transfected indicated constructs in the FLP143 and SH1_7 cells before (−) and after (+) 48 h doxycycline induction. SV40 promoter is used as control. All the constructs express the firefly luciferase gene under the control of a HSV-TK promoter. The transcripts are detected as in (C). The left panel corresponds to a representative northern-blot signal, and the right panel is the normalized quantification of two biological replicates northern blots. The error bar corresponds to the standard deviation of the firefly normalized photostimulated luminescence signal (PSL) of the two replicates. The signal of each probe is represented in white for the normal condition (1,4), in gray for ZNF143 over-expression condition (2,5) and in black for ZNF143 knockdown conditions (3,6).

To better investigate the transcriptional auto-regulation mechanism, we used the heterologous luciferase reporter system already used to define the ZNF143 promoter (Figure 2B). In this system the minimal ZNF143 promoter proximal region (−73/+234) including the TSS1 and the TSS2 regions was placed in front of a *Renilla* luciferase gene. To reproduce the endogenous situation, the construct also contains a truncated splicing-competent version of the ZNF143 first intron placed before the luciferase CDS (Figure 4A). We confirmed by 5′ RACE-PCR that this reporter construct mimics the endogenous situation by producing a TSS1 spliced transcript and a TSS2 transcript initiated in the intron (Figure 4A). We performed substitutions in the SBS/B and C core sequences (CCA to TTG for mut SBS/B− and CCCA to AAAC for mut SBS/C−) that abolish the binding *in vitro* (Figures 4B and 2E). Similar substitutions were known to be detrimental for the formation of ZNF143-DNA complex *in vitro* (29). The SBS/B and C sites were also replaced by the canonical ZNF143-binding motifs (mut SBS/B+ and mut SBS/C+) (Figure 4B) (42). In addition to the SBS mutations, we introduced individual substitution mutations upstream of the SBS/B (Mut 1, position +55/+62 in Figure 4B) and upstream of the SBS/C (Mut 2, position +81/+92; mut 3, position +93/+105; mut 4, position +118/+122; mut 5, position +126/+133; Figure 4B) in the region conserved in vertebrate genomes (Figure 2C). No mutations have been introduced in positions +106/+117 so as not to disrupt the intron splicing mechanism. We followed the TSS1 and TSS2 transcripts in normal and ZNF143 over-expression conditions by northern blot (Figure 4C). We used specific probes (Figure 4A) recognizing the TSS1 transcript (probe 1) or the TSS2 transcript (probe 2). A probe recognizing the firefly luciferase transcript was used to normalize the signal and a GAPDH specific probe was used as a loading control. The results obtained with wild-type (WT) construct show that the over-expression of ZNF143 causes a loss of TSS1 transcript and an increase of the TSS2 transcript level (Figure 4C, compare lanes 1 and 2). This switch of initiation is not affected by the five mutations (mut 1 to mut 5) performed in the conserved blocks (Figure 4C, lanes 3–12, take into account firefly normalization). While the SBS− (SBS/C− and SBS/B−) mutations result in no variation of the TSS1 transcript level after induction (lanes 13 and 14), the SBS+ mutation (high-affinity canonical ZNF143-binding sites: SBS/C+ and SBS/B+) completely abolish the expression of the TSS1 (lanes 15 and 16). This shows that the ZNF143 binding on low-affinity SBS/B and SBS/C sites is essential for the down-regulation of the TSS1 after induction. The TSS2 increased expression following induction is affected by the SBS− mutation. Indeed, the northern blot shows a TSS2 signal decreased by 50% compared to the WT construct (Figure 4C, lanes 13 and 14, take into account firefly normalization). However, while the high-affinity SBS sites (mut SBS+) result in an elevated expression of TSS2, the response to induction is abolished. In this condition, the TSS2 transcript is constantly expressed independently from the ZNF143 over-expression (Figure 4C, lanes 15 and 16). Taken together, the results show that the TSS switch mechanism from TSS1 to TSS2 following the induction is dependent on ZNF143 protein levels and on the low-affinity SBS sites. This observation is confirmed by the higher *in vivo* occupancy observed by ChIP after ZNF143 over-expression (Figure 2G). However, in our experimental conditions, the TSS2 transcription is not fully abolished after induction in SBS− mutant conditions and this will be discussed later.

### The auto-regulation mechanism functions both in ZNF143 over-expression and down-regulation conditions

We recapitulated the previous observations by following the expression of the reporter luciferase (Figure 4A) gene in induced or non-induced FLP143 cells. Individual (mut SBS/C, mut SBS/B) or simultaneous mutations of the SBS motifs (mut SBS−) in non-induced conditions affect slightly the luciferase activity (Figure 4D, left panel). In cells over-expressing ZNF143, the luciferase activity of the WT construct dropped to 20% of the level of non-induced cells. On the other hand, it is only reduced to 40% and 33% of the non-induced signal with the individual mutations in SBS/C (mut SBS/C−) or SBS/B (mut SBS/B−) motifs, respectively. Remarkably, the simultaneous mutation of both the SBS/C and SBS/B (mut SBS− in Figure 4D, left panel) sites almost completely abolish the decrease in luciferase activity in induced conditions. Furthermore, the luciferase activity from the SV40 promoter construct used as a control is not affected by ZNF143 over-expression. The above results obtained with the minimal promoter (−73/+234) were fully recapitulated with the largest promoter region (−593/+234) (data not shown).

Next, we used the SH1_7 inducible stable cell line, to follow the luciferase activity of the same constructs in ZNF143 knockdown conditions (Figure 4D, right panel). After the induction with doxycycline, the SH1_7 cells produce shRNA molecules targeting the ZNF143 messenger RNA, resulting in a specific protein knockdown (Figure 4E, left panel). Surprisingly, we did not observe any variation in the ZNF143 mRNA level following induction (Figure 4E, right panel). The luciferase activity of the SV40 construct used as a control is not affected by ZNF143 knockdown (Figure 4D, right). With the WT construct we observed a 50% increase of the luciferase activity reduced to only 20% with the construct containing the simultaneous SBS/C and SBS/B mutations (mut SBS− in Figure 4D).

This ZNF143-mediated down- or up-regulation of the luciferase activity is most likely linked to a variation in the TSS1 and TSS2 transcripts levels. We hypothesized that the transcription of the TSS1 spliced mRNA results in higher luciferase activity than that of the unspliced TSS2 initiated transcript. This difference could be due to their relative abundance or could be intrinsically linked to the mRNA sequence and affect the stability, nuclear export or translation efficiency. We followed by northern blot the two transcripts in normal, ZNF143 over-expression and ZNF143 knockdown conditions. As previously, we used specific probes recognizing the TSS1 transcript (probe 1), the TSS2 transcript (probe 2) and both transcripts (probe 3) (Figure 4A). The raw northern-blot signal for the WT ZNF143 and the SV40 promoter is shown in Figure 4F (left panel). There is no detectable TSS1 and TSS2 signals in the SV40 control construct (Figure 4F, lanes 4–6). To be compared, all the specific signals were quantified and normalized to the firefly and GAPDH levels (Figure 4F, right part). As expected, after ZNF143 over-expression, the TSS1 level drops down drastically, while the TSS2 increases significantly (lanes 1 and 2). On the other hand, in ZNF143 knockdown conditions, we observed a decrease in TSS2 level and an increase of TSS1 (lanes 1 and 3). The whole *Renilla* transcript level stays relatively stable after ZNF143 over-expression and increases noticeably after ZNF143 knockdown (Figure 4F, probe 3).

The northern-blot analysis endorsed the expected results (Figure 4D) and validates the hypothesis for an auto-regulatory mechanism of ZNF143 expression mediated by a TSS switch that depends on the low or high protein level.

### ZNF143 levels fluctuate during cancer, development and differentiation

**Figure 5.**
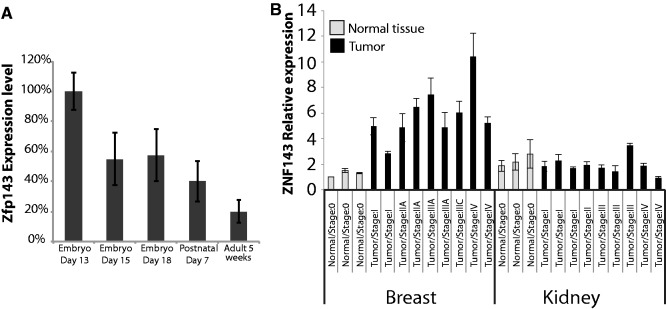
ZNF143 level is up- or down-regulated during development, cancer and differentiation. **(A)** qPCR arrays for ZNF143 gene expression analysis were performed on multiple tissues during mouse development. The bar chart for each developmental stage (embryo, post-natal and adult) corresponds to the mean expression in various tissues (see Supplementary Figure S5). The error bar corresponds to the standard deviation of the ZNF143 relative expression measured in all the tissues at the same developmental stage (Figure S5A). The ZNF143 expression in each tissue corresponds to the mean of two replicates, normalized against GAPDH levels. **(B)** qPCR array for ZNF143 gene expression analysis performed on a panel of human breast and kidney tumor at different stages versus normal tissues. Error bars are standard deviation from two replicates. The ZNF143 expression was normalized against ß-actin levels.

In our previous work, we showed that ZNF143 could be important for rapid cell proliferation (22). In this regard, we wanted to inspect ZNF143 gene expression levels in highly proliferative cells like in tumors and developmental tissues. We first followed by RT-qPCR the ZNF143 transcript level in several mouse tissues from 13, 15 and 18 days embryos, from 7 days post-natal mice and 5 weeks adult. The over whole ZNF143 level is decreasing progressively with the increasing developmental stage (Figure 5A; Supplementary Figure S5A). The 5 weeks adult mice have an over whole 80% lower ZNF143 expression level than the 13 days embryo (Figure 5A). ZNF143 expression is also increased in certain human tumors compared to normal tissues (Figure 5B). ZNF143 levels are rapidly increasing with the breast tumor developmental stage, reaching a 10 times over-expression in cancer stage IV compared to normal tissues. A similarly higher expression of ZNF143 was observed in liver and lung tumors (Supplementary Figure S5B). Instead, in kidney tumors (Figure 5B) as well as in ovary and prostate tumors (Supplementary Figure S5B), ZNF143 expression stays constant compared to normal tissues.

These observations show that in some situations ZNF143 levels fluctuate within a high range without being auto-regulated. Endogenous ZNF143 mRNA levels can even reach a 10-fold increased expression like in breast cancer. High ZNF143 expression seems to be associated with fast-proliferative cells like during the embryonic development or in tumors. Instead, differentiated and normal tissues seem to require a lower ZNF143 expression.

## DISCUSSION

We previously showed that the transcription factor ZNF143 is involved in the control of genes related to rapid cell proliferation (22). Such a factor regulating cell growth processes must be tightly regulated to maintain the correct function of the cells.

We observed that following ZNF143 artificial over-expression there is a decreased expression of the endogenous gene. This down-regulation is directly linked to the DNA-binding specificity of the ectopically produced ZNF143 protein. We showed that this specific down-regulation is reversible and correlates with an increased ZNF143 occupancy on its own promoter region. In addition to the canonically initiated ZNF143 transcript (TSS1 transcript), we identified an alternative ZNF143 transcript (TSS2 transcript) initiated downstream of the TSS1. The balance between the canonical TSS1 and the alternative TSS2 usage is responsible for the ZNF143 transcriptional auto-regulation mechanism. Both the TSS1 and TSS2 initiated transcripts have the same coding potential but differ by their 5′ UTR. The most abundant TSS1 transcript has a short 5′ UTR and permits an efficient ZNF143 protein production. On the other hand, the less abundant TSS2 transcript has a long 5′ UTR originating from alternative non-coding exons located in the canonical intronic region and is poorly translated.

The ZNF143 transcriptional auto-regulation mechanism is protein-dependent, since by treating the FLP143 cells with cycloheximide, we abolished the down-regulation of ZNF143 (data not shown). We did not detect any anti-sense transcript and did not observe any effect on ZNF143 auto-regulation after knocking down the exosome complex (data not shown). A mechanism involving a TSS switch and that is responsive to ZNF143 protein level was, in our opinion, the only plausible hypothesis that we confirmed using an heterologous reporter system.

The TSS switch mechanism is dependent of ZNF143 low-affinity binding sites that are not required for the activity of the ZNF143 promoter. In the human genome, these binding sites are located downstream of the ZNF143 promoter in a highly conserved non coding region of 98 bp exhibiting 100 and 71% identity with the orthologous regions of the mouse and zebrafish genomes respectively. Such conservation of a genomic non-coding region is highly indicative of an important transcriptional mechanism conserved in all vertebrates (43). The non-canonical nature of the ZNF143-binding sites is critical for the control mechanism. Indeed, the conversion of the SBS/B and C sites into canonical sites removes totally the regulation control and the transcription is initiated on the TSS2 independently of the ZNF143 protein level. Following the mutation impairing the *in vitro* ZNF143 binding on SBS/B and C sites, the TSS1 transcription is not anymore responsive to the ZNF143 over-expression in contrast to the TSS2 transcription that is still slightly efficient. This could be explained by a weak ZNF143 binding on mutated SBS/C and SBS/B sites *in vivo* that would affect only the TSS2 transcription. We cannot exclude that the SBS/A site could be also bound by ZNF143 *in vivo* and participate in the TSS2 transcription.

Nevertheless, the non-canonical SBS-binding sites are *per se* sensors for ZNF143 protein level and constitute an element that we define as a ‘sensor platform’. This control region is located adjacent to the 3′-part of the promoter. Besides the sensor platform, the promoter is recognized by other transcription factors distinct from ZNF143. We identified binding sites validated by ChIP-Seq for GABPA (35) and NFYA (44) transcription factors upstream of the canonical TSS. These sites are essential for the general promoter activity but have no impact on ZNF143 auto-regulation (data not shown).

**Figure 6.**
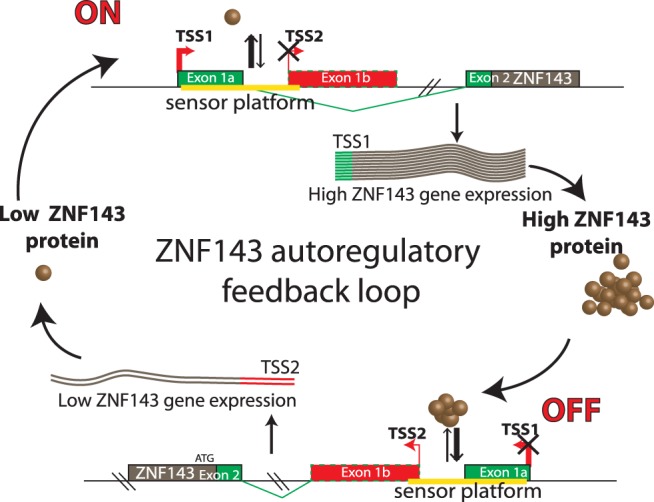
A model for ZNF143 auto-regulatory feedback loop. The ZNF143 gene expression control relies on the balance between TSS1 and TSS2 transcription initiation. This balance is adjusted by a sensor platform containing non-canonical SBS sites (represented in yellow). The low occupation by ZNF143 of the sensor platform leads to the stimulation of the transcription of the TSS1 transcript and a decreased transcription of the TSS2 transcript. In ZNF143 over-expression conditions, the increased occupation of the sensor platform restricts the expression of TSS1 transcript in favor of the weakly expressed TSS2 transcript.

Our results allowed us to propose a model for a ZNF143 auto-regulation mechanism that maintains its adequate level in the cell (Figure 6). In ZNF143 protein over-expression conditions, the increased occupancy of the ‘sensor platform’ results in the production of the TSS2 transcript. Lower ZNF143 gene expression combined with a poor translation efficiency of the TSS2 transcript results in low protein production to equilibrate the level of the factor in the cell. When the ZNF143 protein is under-expressed, the lowered occupancy on the sensor platform results in high TSS1 transcription. Thus, this leads to high protein production to equilibrate the transcription factor shortage in the cell.

Interestingly, we noticed that following the induction of our SH1_7 cell line, we could observe only a knockdown of the protein, while the ZNF143 mRNA remained stable (Figure 4E). This observation could be reasonably explained by the above-mentioned ZNF143 auto-regulatory mechanism.

It is noteworthy to point out that ZNF76 over-expression also induced ZNF143 down-regulation. This observation was not surprising since the two proteins have the same DBD (31) and occupies the same binding site *in vivo* (22). Moreover, ZNF76 transcription is not affected by itself neither by the ZNF143 protein. We observed that ZNF76 protein level is very low in HeLa and 293 T-rex cells and is detectable by western blot only after immunoprecipitation from 10^8^ cells (data not shown). If both proteins have redundant functions, we hypothesize that ZNF76 would have been maintained during evolution as a safety measure to keep a basal ZNF143 transcription factor level in the cell.

Despite its auto-regulatory feedback loop, we observed that ZNF143 level is fluctuating in tumors and development. In stage IV breast cancer, for example, ZNF143 is 10 times more expressed than in normal tissue. In these situations we could speculate that ZNF143 escapes somehow from its auto-regulation. This evasion could be mediated by external stimuli that have been already reported to affect ZNF143 expression, such as lactogenic hormones (45), insulin-like growth factor-1 (46) or the calcium levels affect ZNF143 expression (47). In this respect, a putative NFAT5-binding site located in the 98 bp highly conserved element covering the sensor platform could be likely related to the ZNF143 calcium dependent regulation. Nonetheless, how the transcriptional auto-regulation is switched on or off, need to be further investigated.

Around 35% of human coding genes contain introns in the 5′ UTR, in particular genes with regulatory roles (48). Alternative promoter usage and alternative 5′ UTRs are well known to be involved in gene expression regulation (1). Nevertheless, as far as we know, there is no example of transcription factor auto-regulation involving a TSS switch mechanism relaying on a sensor platform containing non-canonical binding sites to generate transcripts with alternative 5′ UTR.

**Figure 7.**
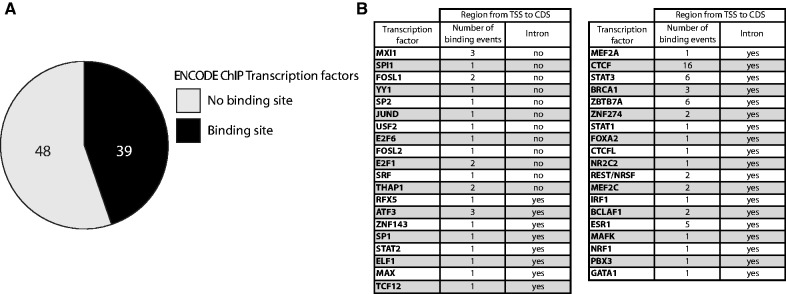
Transcription factors potentially subjected to an auto-regulation. **(A)** Pie chart depicting the proportion of the 87 sequence-specific transcription factors from ENCODE ChIP-seq experiments (49) having a binding event in their own 5′ non-coding region (5′ non-coding exon and introns located before the CDS). **(B)** Table summarizing the number on binding events found in the 5′ non-coding region of the 39 sequence-specific transcription factors. For each factor the presence or absence of at least one intron in the 5′ non-coding region is mentioned.

Having discovered that ZNF143 level is maintained in the cell through an auto-regulatory feedback loop involving a TSS switch, we scrutinized the publicly available ENCODE ChIP-seq data to reveal if this mechanism could be also used by other transcription factors. Of the 118 ENCODE transcription factors (49) we selected only the sequence-specific transcription factors and as for ZNF143, we were interested in the regulatory region located between the TSS and the CDS, including the eventual non-coding exons and introns. Using UCSC table browser (50) we extracted the ENCODE ChIPseq signal track and overlapped with the transcription factors regulatory regions. For each factor, the cluster score represents the strength of the ChIP-seq signal that is influenced by the affinity of the factor to one or more binding sites present in the cluster. The resulting table is available in supplementary material (Supplementary Table S2). Surprisingly, of the 87 sequence-specific transcription factors, we found that 39 of them have at least one binding event in their own regulatory region (Figure 7A and B). Among these factors, 12 have no intron in their regulatory region while 27 contain at least an intron in their 5′ UTR (Figure 7B). As for ZNF143 some of these factors, such as CTCF and NRSF, have a non-coding first exon and a binding site in their first intron. We hypothesize that these 39 transcription factors could be subjected, like ZNF143, to an auto-regulatory feedback loop.

To summarize, we identified in this study a novel auto-regulatory mechanism that involves a switch of TSSs and that relies on non-canonical binding sites usage. We uncovered this mechanism using ZNF143 gene as a model, but many more transcription factors could be subjected to such a transcriptional auto-regulatory feedback loop using alternative TSS and low-affinity binding sites to maintain their right level in the cell.

## SUPPLEMENTARY DATA

Supplementary Data are available at NAR Online.

## FUNDING

Université de Strasbourg; Centre National de la Recherche Scientifique; Ministère de l'Enseignement Supérieur et de la Recherche (to R.P.N.); Association pour la Recherche contre le Cancer (to R.P.N.); Ligue Contre le Cancer (CCIR-GE). Funding for open access charge: CNRS and Ligue Contre le Cancer (CCIR-GE).

*Conflict of interest statement*. None declared.

## Supplementary Material

Supplementary Data
