# Supplementary material for: Transcription factor abundance controlled by an auto-regulatory mechanism involving a transcription start site switch
Source: Nucleic Acids Res. 2013 Nov 14;42(4):2171–84. doi: 10.1093/nar/gkt1136 (PMC3936768; doi:10.1093/nar/gkt1136)
Supplement: Supplementary Data [file supp_42_4_2171__index.html]

Transcription factor abundance controlled by an auto-regulatory mechanism involving a transcription start site switch — Transcription factor abundance controlled by an auto-regulatory mechanism involving a transcription start site switch — Supplementary Data 

# Transcription factor abundance controlled by an auto-regulatory mechanism involving a transcription start site switch

## Supplementary Data

files

**Files in this Data Supplement:**

- Supplementary Data - pdf file
- Supplementary Data - xlsx file
- Supplementary Data - xlsx file
