# Supplementary material for: Transcription factor abundance controlled by an auto-regulatory mechanism involving a transcription start site switch
Source: Nucleic Acids Res. 2013 Nov 14;42(4):2171–84. doi: 10.1093/nar/gkt1136 (PMC3936768; doi:10.1093/nar/gkt1136)
Supplement: Supplementary Data [file supp_gkt1136_nar-02079-x-2013-File009.pdf]

SUPPLEMENTARY MATERIAL AND METHODS

Constructs

Constructs used for the generation of the FLP143A5, FLP143-HA, FLP_THAP11-HA, FLP76 stable cell lines are described in (22). The construct used for the generation of the FLP143-EGR2 stable cell line, expressing a chimeric ZNF143 protein (ZNF143-EGR2), was obtained by replacing the DNA binding domain of ZNF143 by the DNA binding domain of EGR2.

Briefly, the XhoI (+705 from the ATG) and EcorV (+1339 from the ATG) restriction sites were added, by site directed mutagenesis, on either side of the sequence coding the DNA binding domain of ZNF143, using the primer pairs GM5/GM6 and GM7/GM8. The EGR2 DNA binding domain, flanked by XhoI and EcoR1 sites, was amplified by PCR from the pBRN3-ZNF143-Krox-20 plasmid (31) using the primers GM9 and GM10. The DNA binding domain of ZNF143 was replaced by the DNA binding domain of EGR2 using the XhoI and EcorV sites. The final construction was subcloned into pcDNA5/FTR/TO using BamH1 site, and the resulting pGMC plasmid was used for the stable cell line generation using the Flp-In™ T-Rex™ 293 system (Invitrogene) as described in (22). The chimeric ZNF143-EGR2 protein is 535 residues long and contains 3 zinc fingers in its DBD, while the ZNF143 protein has 638 residues and contains 7 zinc fingers. The sequences of all primers are available in supplementary table S1.

The ZNF143 cDNA clone H04D117B14 (TSS2) was obtained from the RIKEN full-length enriched human cDNA library (EST: HY033867 and HY218615). The canonical ZNF143 TSS1 cDNA clone (IMAGE: IMAGp998J186881Q) was cloned in pcDNA 3.1+ (Invitrogen) expression vector on EcoR1 and XhoI sites. The alternative ZNF143 TSS2 cDNA clone (H04D117B14) was cloned in pcDNA 3.1+ on NheI and BamHI sites.

Promoter constructs: All the constructs are derived from parental pNG43 construct which is a derivate of the psicheck-2 (Promega) vector generated by insertion of the ß-globin 3'UTR into the XhoI and NotI sites of psicheck-2. The 3'UTR of ß-globin was amplified from the pBS2415 vector with the PN208 and PN209 oligos. The EVA5 construct (-593 in Figure 2B) was obtained by replacing the SV40 promoter and the chimeric intron (KpnI/NheI fragment) from pNG43 by a KpnI/NheI fragment containing the promoter region of ZNF143 (593 bp upstream of the TSS) associated to a shortened ZNF143 first intron. The shortened intron contains the 5' (+1/+234) and 3' parts (+10170/+10344) of the ZNF143 first intron linked together with an AgeI site. EVA6, EVA7, pNG107 and EVA8 constructs were obtained as EVA5 but by the use of promoter region of ZNF143 truncated at positions -390, -196, -76 and -25 respectively (Figure 2B). The 5' truncated promoters and the 5' part of the first intron were amplified with the following primers pairs: PN279/PN637 (-593: EVA5); EV1/PN637 (- 390: EVA6); EV2/PN637 (-196: EVA7); PN617/PN637 (-76: pNG107); EV3/PN637 (-25: EVA8). The 3' part of the ZNF143 first intron was amplified from genomic DNA using PN638/PN639 primers pair. The primer sequences are available in the supplementary Table S1. The mut SBS/C- construct (pNG108) was obtained from pNG107 by site-directed substitution mutagenesis of the CCCA at positions +136/+139 by AAAC using PN355 and PN356 oligonucleotides. The mut SBS/B- construct (pNG109) was obtained from pNG107 by mutagenesis of the TGG at positions +75/+77 by CAA using PN621 and PN622 oligonucleotides. The mut 1 (EVA13), mut 2 (E508), mut 3 (EVA15), mut 4 (pNG102) and mut 5 (EVA16) constructs were obtained from EVA5 by substituting respectively the TTTCCTGT by gggaagtg at position +55/+62 (using the EV5/EV6 primers), the GACGAAGGAATT by tcatccccttgg at position +81/+92 (using the EV26/EV27 primers), the GTTGGAAAATTTT by tggttcaccgggg at position +93/+105

(using the EV8/EV9 primers), the TATAT by gcgcg at position +118/+122 (using the PN623/PN624 primers) and the TGTGTTTT by gtggggtg at position +126/+133 (using the EV10/EV11 primers). The mut SBS+ (E509) construct was obtained from EVA5 by substituting the CCTGGTGCATGGTGGTCG by cgcggggcatgctgggag at position +63/+80 (using the EV22/EV23) and TGTGCATTATT by tacccagcatgccccgcg at position +141/+151 (using the EV24/EV25 primers). Substitution mutants were generated with the QuikChange II XL kit (Stratagene) and verified by DNA sequencing.

Antibodies

Antibodies used in ChIP analyses were as follows: ZNF143, rabbit polyclonal raised against a C-terminal epitope of ZNF143 (22); ZNF76, mouse monoclonal (Santa Cruz Biotechnology, cat. #sc-81147); HA, 12CA5 antibody, (Roche Applied Science), RNA polymerase II CTD (Abcam, ab5408), H3K4me3 (Abcam ab12209), H3K9me3 (Abcam ab8898). Western blot analysis was performed with the antibodies above or antibodies against α-tubulin, mouse monoclonal (Sigma, T6557), TBP mouse monoclonal 3G3 antibody, ZNF143 (Abcam ab58168).

SUPPLEMENTARY FIGURE LEGENDS

Figure S1: ZNF143 is involved in a reversible auto-regulatory negative feedback loop. (A) Northern blot analysis of the endogenous ZNF143 down-regulation following the overexpression of the ZNF143 transgene. Enriched Poly(A)+ RNA from FLP143 cells non (0h) or induced 24 hours (24h) was used to follow the endogenous ZNF143. On the top, schematic representation of the ZNF143 mRNA. The probe 1 complementary to the CDS detects the products of endogenous ZNF143 and transgene. The probe 2 complementary to the 3'UTR detects the endogenous ZNF143. The ribosomal RNA 28S is shown as loading control. **(B)** Western blot performed on total protein extracts from FLP143 cells at different times points (in hours) after induction of the ZNF143 over-expression. The inductor (doxycycline) was washed out after 6 hours. The arrows indicate the ZNF143 protein. The TBP protein is used as loading control. **(C)** ZNF143 gene expression measured by RT-qPCR on total RNA extracted from FLP143 cells at different time points (in hours) post-induction of the ZNF143 over-expression. The inductor (doxycycline) was washed out after 6 hours. The relative expression of the endogenous ZNF143 mRNA (endo ZNF143) was followed using primers specific to the 5'UTR. The total ZNF143 (endogenous and overexpressed) was followed with primers targeting the coding sequence. The relative expression was normalized to both the TBP and UBC gene expression levels.

Figure S2: ZNF143, TBP and TAF1 proteins bind the promoter of the ZNF143 gene. (A) UCSC genome browser view of a ZNF143 ChIP-seq peak (22) located on its own

promoter region in mouse embryonic stem cells (mESC). **(B)** UCSC genome browser view of TBP and TAF1 peaks (34) in ZNF143 promoter in HeLa cells

Figure S3: Histone modification marks, ZNF143 and Pol II occupancy on ZNF143 gene promoter region. ChIP-qPCR experiment on non-induced and 24h induced FLP143 cells. On the top, schematic representation of the ZNF143 gene with positions relative to the transcription initiation site (+1) of the qPCR primer pairs. ChIP was performed with antibodies against ZNF143, H3K4me3, Pol II and H3K9me3. A ChIP control was performed using non-specific antibodies (anti-HA). ChIP enrichment was evaluated by qPCR using specific primers targeting ZNF143 promoter regions, (-150 bp, +300 bp, + 800 bp). A region located 1 kb downstream of ZNF143 gene (+66 kbp) is used as negative control. The GAPDH promoter is used as a positive control for H3K4me3 and Pol II. Primers amplifying a satellite 2 region (SAT2) are used as a positive control for H3K9me3 marks. The enrichment is represented in percentage of input DNA (% of input).

Figure S4: Alternative transcription start site usage generates two ZNF143 transcripts with distinct 5'UTRs. (A) Schematic representation of the ZNF143 gene and TSS identified by 5'RACE PCR in total RNA from induced and non-induced FLP143 cells. The canonical transcription initiation site is represented with an arrow (+1 Refseq). The first non-coding exon sequence is highlighted in green and the sequence of the first intron in yellow. The sequences identified by sequencing the 5'RACE-PCR products are aligned under the reference transcript. The first nucleotide is indicated in red. The first set of sequences identified corresponds to the canonical transcripts (TSS1), initiated in a window located in the first canonical non-coding exon. The second set of

sequences initiated in the beginning of the first intron corresponds to the alternative TSS2 transcript. **(B)** UCSC genome browser view of the ZNF143 expressed sequence tags (EST), initiated from two different transcriptional start sites (TSS1 and TSS2). **(C)** Schematic representation of the ZNF143 gene and of the two alternative transcripts (TSS1 and TSS2). A UCSC genome browser view shows in blue the canonical TSS1 transcript containing 16 exons and the alternative transcript TSS2 of 19 exons in black. The two transcripts have the same CDS, represented in dark blue. The 5'UTR of the TSS1 transcript is represented in red and that of the TSS2 transcript in dark green. The sequences of the two 5'UTRs are shown in green for the TSS2 and in red for the TSS1. **(D)** Relative abundance of endogenous TSS1 (ZNF143 TSS1); endogenous TSS2 (ZNF143 TSS2); both TSS1 and TSS2 transcripts (ZNF143 endo); total ZNF143 (ZNF143 total) transcripts, in FLP143 cells before induction. The abundance of the different transcripts is evaluated by qPCR in terms of mean quantification cycles (Cq) using the same detection threshold.

Figure S5: ZNF143 levels are up- or down-regulated during development and cancer. **(A)** qPCR arrays for ZNF143 gene expression analysis performed on multiple tissues during mouse development. The ZNF143 expression was measured in tissues of 13, 15 and 18 days embryos; 7 days postnatal mice and 5 weeks adult mice. Error bars are standard deviation from two replicates. The ZNF143 expression was normalized against GAPDH levels. **(B)** qPCR array for ZNF143 gene expression analysis in colon, liver, lung, ovary, prostate and thyroid tumors at different stages versus normal tissues. Error bars are standard deviation from two replicates. The ZNF143 expression was normalized against ß-actine levels.

Figure S1

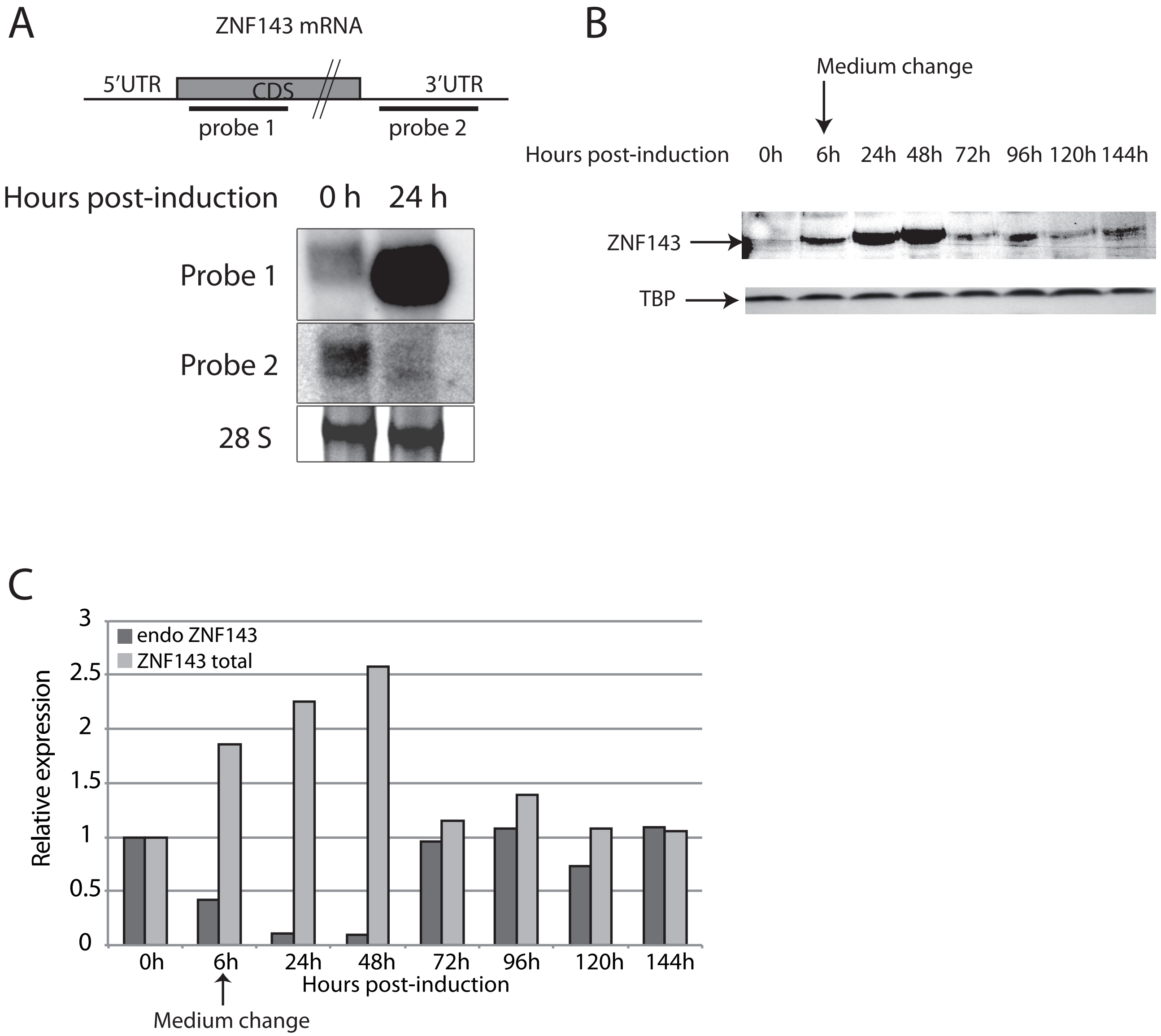

A
ZNF143 mRNA
5′UTR
CDS
3′UTR
probe 1
probe 2
Hours post-induction
0 h
24 h
Probe 1
Probe 2
28 S
B
Medium change
Hours post-induction
0h
6h
24h
48h
72h
96h
120h
144h
ZNF143
TBP
C
endo ZNF143
ZNF143 total
Relative expression
3
2.5
2
1.5
1
0.5
0
0h
6h
24h
48h
72h
96h
120h
144h
Hours post-induction
Medium change

Figure S2

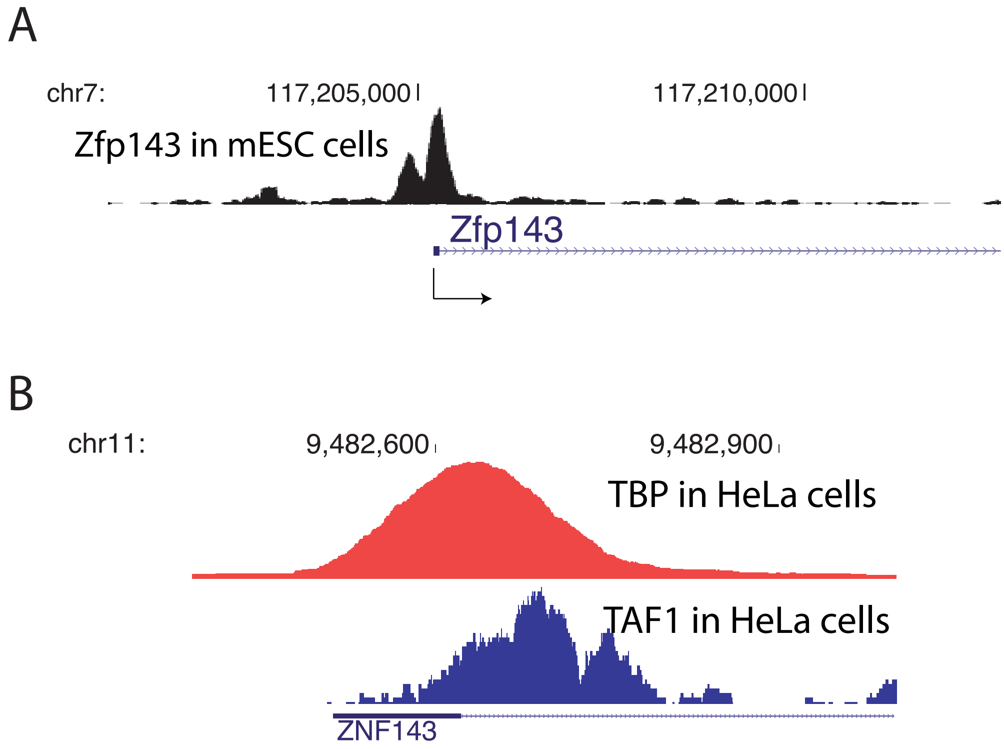
A
chr7:
117,205,000
117,210,000
Zfp143 in mESC cells
Zfp143
B
chr11:
9,482,600
9,482,900
TBP in HeLa cells
TAF1 in HeLa cells
ZNF143

Figure S3

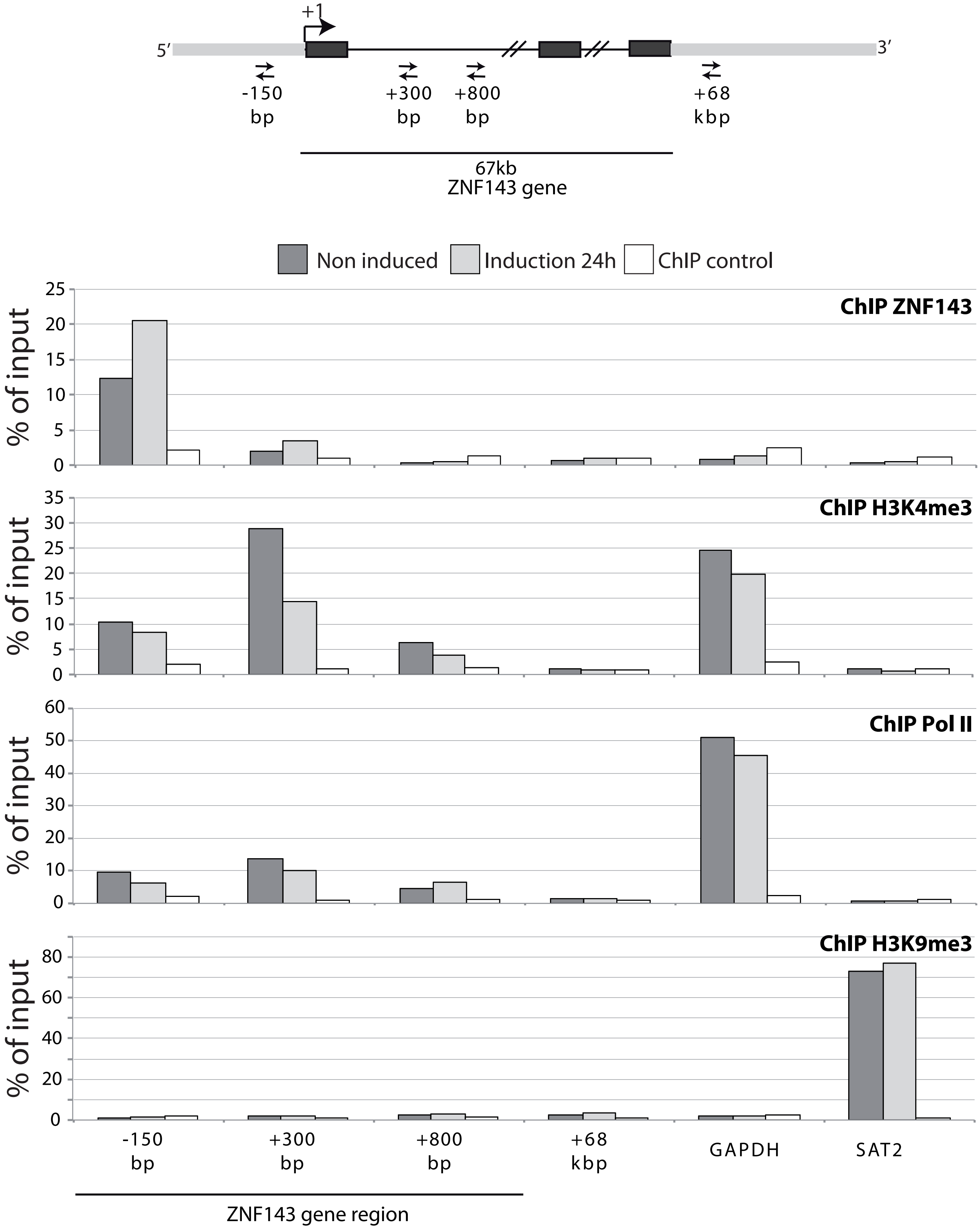
+1
5′
3′
-150 bp
+300 bp
+800 bp
+68 kbp
67kb
ZNF143 gene
Non induced
Induction 24h
ChIP control
ChIP ZNF143
ChIP H3K4me3
ChIP Pol II
ChIP H3K9me3
% of input
25
20
15
10
5
0
35
30
60
50
40
80
-150 bp
+300 bp
+800 bp
+68 kbp
GAPDH
SAT2
ZNF143 gene region

Figure S4

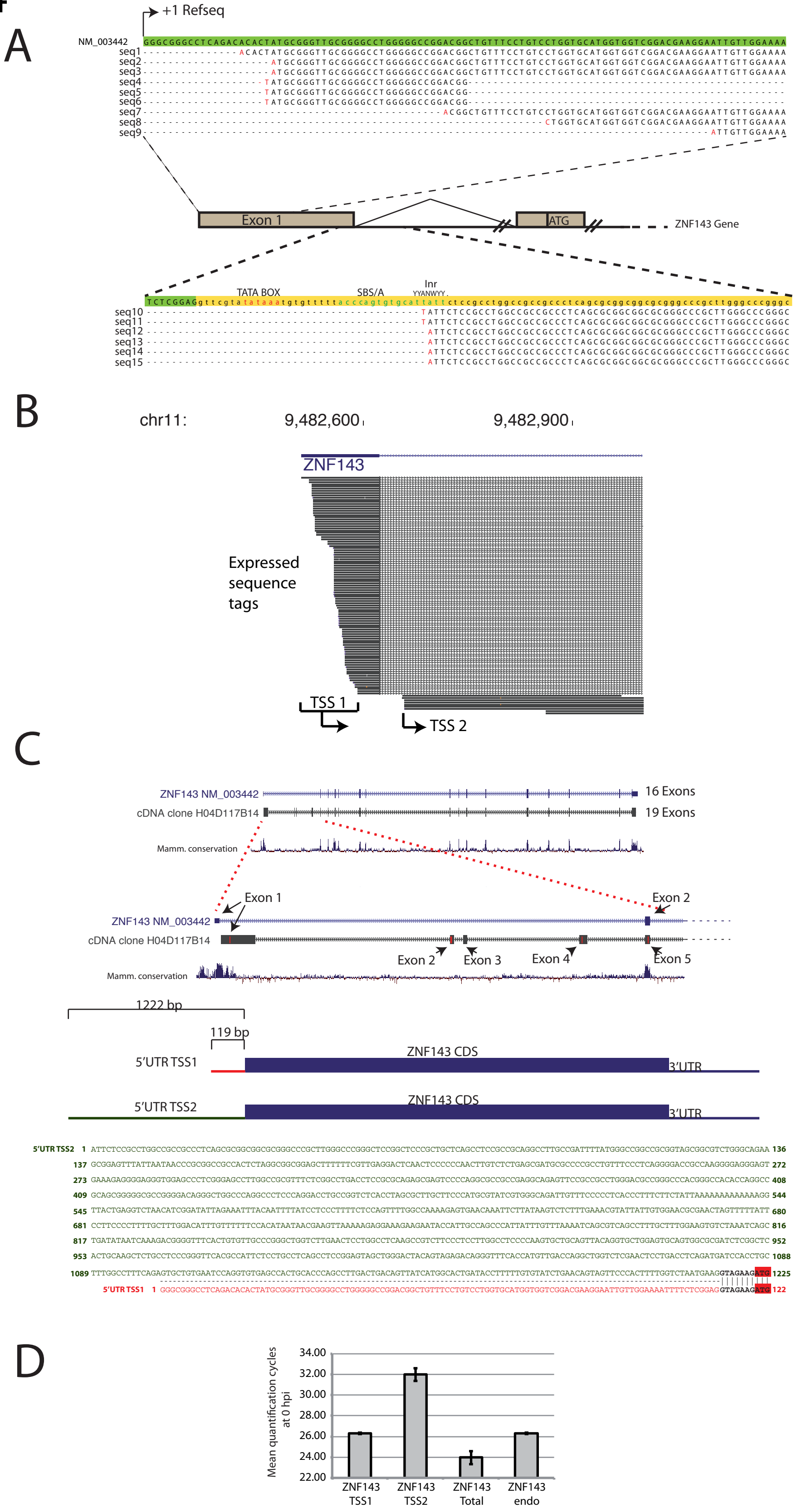
A
+1 Refseq
NM_003442
seq1
seq2
seq3
seq4
seq5
seq6
seq7
seq8
seq9
Exon 1
ATG
ZNF143 Gene
TATA BOX
SBS/A
Inr
seq10
seq11
seq12
seq13
seq14
seq15
B
chr11:
9,482,600
9,482,900
ZNF143
Expressed
sequence
tags
TSS 1
TSS 2
C
ZNF143 NM_003442
16 Exons
cDNA clone H04D117B14
19 Exons
Mamm. conservation
Exon 1
Exon 2
ZNF143 NM_003442
cDNA clone H04D117B14
Exon 2
Exon 3
Exon 4
Exon 5
Mamm. conservation
1222 bp
119 bp
5'UTR TSS1
ZNF143 CDS
3'UTR
5'UTR TSS2
ZNF143 CDS
3'UTR
5'UTR TSS2
5'UTR TSS1
D
Mean quantification cycles
at 0 hpi
34.00
32.00
30.00
28.00
26.00
24.00
22.00
ZNF143
TSS1
ZNF143
TSS2
ZNF143
Total
ZNF143
endo

Figure S5

A

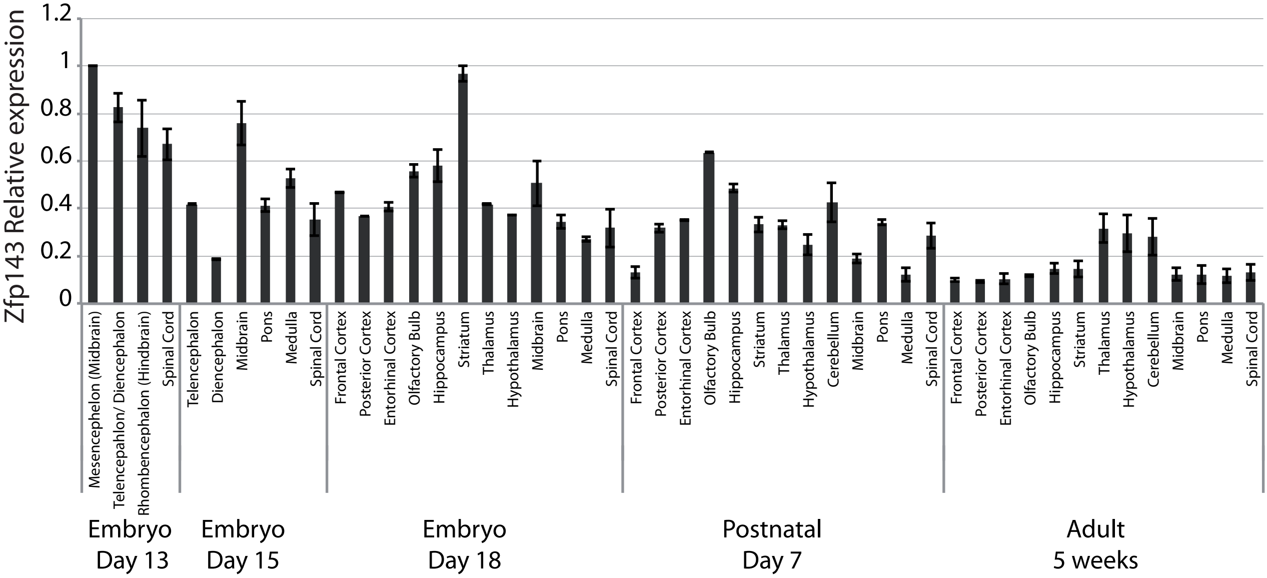
Zfp143 Relative expression
1.2
1
0.8
0.6
0.4
0.2
0
Mesencephelon (Midbrain)
Telencepahlon/ Diencephalon
Rhombencephalon (Hindbrain)
Spinal Cord
Telencephalon
Diencephalon
Midbrain
Pons
Medulla
Spinal Cord
Frontal Cortex
Posterior Cortex
Entorhinal Cortex
Olfactory Bulb
Hippocampus
Striatum
Thalamus
Hypothalamus
Midbrain
Pons
Medulla
Spinal Cord
Frontal Cortex
Posterior Cortex
Entorhinal Cortex
Olfactory Bulb
Hippocampus
Striatum
Thalamus
Hypothalamus
Cerebellum
Midbrain
Pons
Medulla
Spinal Cord
Frontal Cortex
Posterior Cortex
Entorhinal Cortex
Olfactory Bulb
Hippocampus
Striatum
Thalamus
Hypothalamus
Cerebellum
Midbrain
Pons
Medulla
Spinal Cord
Embryo Day 13
Embryo Day 15
Embryo Day 18
Postnatal Day 7
Adult 5 weeks

B

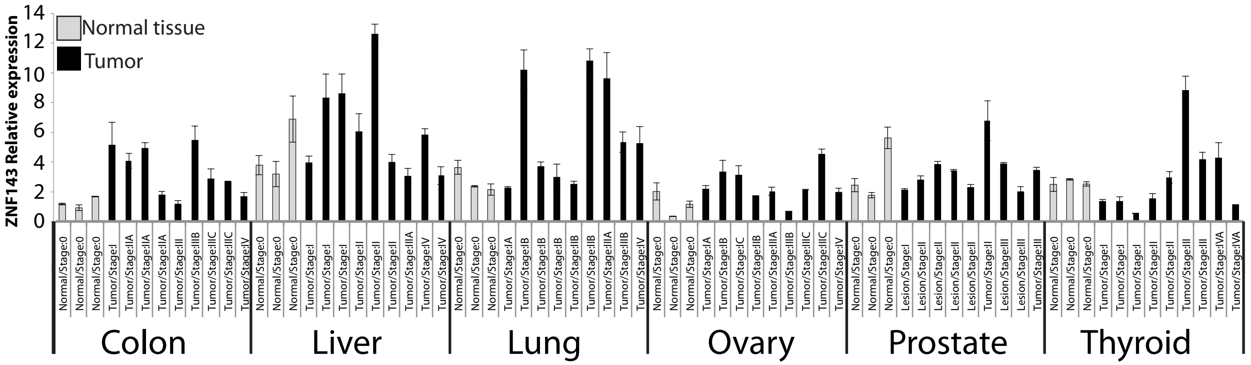
ZNF143 Relative expression
Normal tissue
Tumor
14
12
10
8
6
4
2
0
Colon
Liver
Lung
Ovary
Prostate
Thyroid
